# Billy Mackenzie: Sometimes Wild, Often Lonely

**DOI:** 10.1002/hup.70021

**Published:** 2025-10-06

**Authors:** David Baldwin

**Affiliations:** ^1^ University Department of Psychiatry University of Southampton Southampton UK

William MacArthur (‘Billy’) Mackenzie died alongside an apologetic suicide note, in a garden shed at his father's cottage in Auchterhouse, Scotland, after an overdose of amitriptyline, beta‐blockers and paracetamol. He was 64 days short of his fortieth birthday. Three weeks earlier, on New Years Eve 1996, he had been admitted to Ninewells Hospital, Dundee, requiring assisted ventilation after consuming a large quantity of prescribed hypnotics. Then, his father had entreated the assessing psychiatrist to transfer his son to the hospital psychiatry ward, but Billy had maintained the overdose was an ‘accident’, so he was instead discharged home with a recommendation to start antidepressants.

Some 15 years previously, Mackenzie stood at the threshold of international success (Doyle 1998). With his musical collaborator Alan Rankine, as ‘The Associates’, the band had released six singles over the preceding 12 months, to increasing critical acclaim: their mysterious, fragmented lyrics and disconcerting melodic shifts somehow captured both the desolate landscapes and agitated unease of post‐industrial Thatcher‐ravaged Britain. What promised to be an *annus mirabilis* in 1982 saw the band appear repeatedly on BBC's *Top of the Pops* and featured adoringly in glossy teen mags. Highbrow music newspaper journalists described their third album (*Sulk*), released in May, as ‘an elusive butterfly’, yet possessing a ‘timeless majesty’. It stayed high in the album charts for 20 weeks. But in October Mackenzie threw it all away, by declining to perform publicly ‐ so causing the departure of an exasperated Rankine ‐ on the eve of a widely anticipated tour of British concert venues.

Admittedly, from an early age Mackenzie was given to choosing impulsively and acting recklessly, and gushing public praise fitted poorly with his dismissive self‐criticism. He had a track record of doing crazy things: as a teenager, marrying an American presumptive heiress in Las Vegas; whilst recording, consuming so many psychostimulants as to require 3 days' cardiac monitoring in St Mary's Hospital, London. But there were additional factors in this career‐damaging decision. Although an impish grin, flirtatious manner and occasional camp pugnaciousness suggested a cool ease in public encounters, by 1978 he had become troubled by recurring ‘stage fright’ immediately prior to performance. The lyrics to The Associates' best‐known song *Party Fears Two* (a top‐10 single) can be read as a description of debilitating social anxiety and the temporary relief afforded by alcohol consumption. However, it was only after many years that could Mackenzie confide about his experience of disabling panic: by then, it was hard to board flights, or to be separated far from his mother Lily, to whom he was devoted.

The 15 years from *Sulk* should not be regarded simply as a sad, slow decline. A succession of collaborations under the continuing name ‘Associates’ and a sequence of cameo appearances with other musicians reminded audiences of his exceptional voice, which could switch so deftly from baritone to falsetto within a single line. However, subsequent albums (*Perhaps*, 1985; *Wild and Lonely*, 1990) situated their captivating moments within much over‐produced material: and whilst the higher energy tracks became fêted in New York night clubs, the released singles were at most only minor hits. Critics turned, questioning the continuing relevance of the band. Mackenzie had long struggled without the oversight of a financially astute manager and the protection of a personal assistant and when he made such appointments, it was already too late. The ‘band’ was dropped by successive companies due to beyond‐budget recordings but limited commercial success. The Associates album *The Glamour Chase*, first recorded in 1988, was eventually released five years after Mackenzie's death.

Mackenzie had become dogged by a disabling perfectionism, nagging dissatisfaction with his vocal contributions, and creeping bitterness about the music industry apparatus. Marked sensitivity about a visibly receding hairline made him reluctant to appear in public. The first album under his own name (*Outernational*, 1992) somehow submerged his individuality below glacial over‐instrumentation and may have sold only three thousand copies (Doyle 1998). A half‐hearted reunion with Rankine in 1993 produced promising material (described as ‘techno Goth—nightmarish and dark’) but this edginess was not welcomed by record companies during the Brit‐pop nostalgia‐fest of the early 1990s. Mackenzie continued writing new songs with trusted collaborators but seemed unable to settle on an integrative style, veering between piano‐based ballads and guitar‐buzzing rock tracks; and without a contract had no creative outlet. Having never learnt to live within his financial means, his flat was repossessed in April 1995, and Mackenzie was declared bankrupt.

In the early months of 1996 his mother was diagnosed with terminal stomach cancer, and Mackenzie started smoking cigarettes compulsively. When she deteriorated suddenly over one day in July, he rushed back home from Edinburgh but she had passed away before he arrived. Although he would talk about her for hours, family members and friends felt he was unable to express the full extent of his grief. In his last interview, 2 weeks after her death, Mackenzie admitted he had always been a reluctant star and obliquely acknowledged his bisexuality: and talked of his desire to release three albums in quick succession, in disparate genres. Unexpectedly, he was offered a recording contract based on demos of piano ballads, and in October started sessions for *Beyond The Sun*, the final album completed during his lifetime. His last recording, in December, was the vocal to *Pain in Any Language* written with Apollo 440, a Liverpudlian electronic band. By then he had started to fade away. Now living with his father, Mackenzie was depleted by exhausting train journeys between Dundee and London. Friends became concerned about his obvious loneliness, withdrawal, and the brooding belief that he had let his mother down. Mackenzie became reluctant to be left alone, or to leave home with others, and talked about his inability to sleep.

It is easy to imagine the quicksilver tongue of Mackenzie persuading the hospital psychiatrist to allow him home following the hypnotic overdose: and it was a Bank Holiday, when not much happens in hospital. But having recognised Mackenzie as depressed and in need of antidepressant treatment, the subsequent prescription of the ‘loaded gun’ of amitriptyline ‐ with its additional magazine of beta‐blockers ‐ is hard to understand. Comprehensive assessment would have determined that Mackenzie was at considerable risk of further suicide attempts. The high cardiotoxicity of amitriptyline in overdose was long established and widely known (Montgomery et al. 1989) ‐ and indeed remains a cause of concern (Taylor et al. 2024).

The course of events might have differed if an antidepressant was first prescribed when Mackenzie sought treatment for persistent insomnia, before the overdose. Maybe amitriptyline was recommended believing it might facilitate sleep, and a beta‐blocker added thinking it could quell agitation, although many safer alternatives (notably, selective serotonin reuptake inhibitors) were readily available. But the chosen drugs did not work. Insomnia worsened, he lost weight and his sense of humour and told friends that he was experiencing hallucinations. He started to take his leave: he attempted to make financial provisions for his musical collaborators and sang to his frail grandmother in her residential home. The day before his death, Mackenzie had informed his father he was going to Dundee, but he remained in Auchterhouse and may have last been seen alive wandering the fields above his father's home, draped in a duvet.

This is both a lament and a tribute. Mackenzie may have been happiest in his cherished pursuit of breeding and raising whippet dogs, and he often joshed that he and his musical output would soon be forgotten. But when *Beyond the Sun* was released 9 months after his death, it received multiple accolades, including ‘essential’ (Daily Telegraph) and ‘perfectly beautiful’ (Melody Maker). Profits from its sales were directed to Macmillan Cancer Relief and The Samaritans. Listening to it now, it sounds as if finished rather hastily. Mackenzie needed more time to nurture his songwriting and share his glorious four‐octave vocal range, beside sensitive collaborators. Those gathered at the 10‐year anniversary commemorative concert held in London in March 2007 expressed the commonly held view that he had merely been born a decade too soon. We still need to hear his voice.

## Conflicts of Interest

The author declares no conflicts of interest.

## Data Availability

The author has nothing to report.
